# Energy Substrate Utilization and Body Composition Following Exercise Preconditioning During Alternate-Day Fasting and Concurrent Exercise in Mice

**DOI:** 10.3390/metabo16090618

**Published:** 2026-08-27

**Authors:** Jungyong Lee, Taeho Kim, Deunsol Hwang, Sunghwan Kyun, Inkwon Jang, Nayeon Kim, Kiwon Lim, Hun-Young Park, Insu Kwon, Sung-Woo Kim, Jisu Kim

**Affiliations:** 1 Laboratory of Exercise & Nutrition Mechanisms, Department of Sports Medicine and Science in Graduate School, Konkuk University, Seoul 05029, Republic of Korea; jy4812@konkuk.ac.kr (J.L.); qwer92224@konkuk.ac.kr (T.K.); hds49@konkuk.ac.kr (D.H.); y10345@konkuk.ac.kr (S.K.); inkwon555@konkuk.ac.kr (I.J.); kny010320@konkuk.ac.kr (N.K.); exercise@konkuk.ac.kr (K.L.); parkhy1980@konkuk.ac.kr (H.-Y.P.); insu0115@konkuk.ac.kr (I.K.); kswrha@konkuk.ac.kr (S.-W.K.); 2 Physical Activity and Performance Institute (PAPI), Konkuk University, Seoul 05029, Republic of Korea; 3 Department of Physical Education, Konkuk University, Seoul 05029, Republic of Korea

**Keywords:** exercise preconditioning, alternate-day fasting, concurrent exercise, energy substrate utilization, body composition

## Abstract

**Highlights:**

**What are the main findings?**

- Exercise preconditioning was associated with lower body fat percentage and fat mass, and these differences were maintained after alternate-day fasting and concurrent exercise.
- Exercise preconditioning was associated with significantly higher resting oxygen uptake, carbon dioxide production, respiratory exchange ratio, carbohydrate oxidation, and energy expenditure after the intervention.

**What are the implications of the main findings?**

- Exercise preconditioning may be associated with the maintenance of lower adiposity and distinct substrate-utilization patterns during a subsequent combined intervention.
- Future studies should conduct longer-term human interventions to evaluate the feasibility, adherence, lean-mass preservation, and metabolic benefits of exercise preconditioning in real-world settings.

**Abstract:**

**Background/Objectives:** Alternate-day fasting (ADF) combined with exercise has been suggested as an effective lifestyle strategy for reducing fat mass while preserving lean mass. However, whether exercise preconditioning before ADF plus concurrent exercise affects subsequent body-composition and substrate-utilization responses remains unclear. This study aimed to evaluate the effects of exercise preconditioning during a subsequent ADF plus concurrent exercise intervention. **Methods:** Seven-week-old male ICR mice were assigned to the non-exercise preconditioning (Non-ExPC) or exercise preconditioning (ExPC) group (*n* = 8/group). After the 4-week preconditioning period, both groups underwent 7 days of ADF plus concurrent exercise. Resting energy metabolism was measured during the intervention, and body composition, exercise energy metabolism, muscle and motor function, blood biomarker concentrations, and plasma hormone levels were assessed before and after the intervention. **Results:** Both groups showed significant reductions in body weight, body fat percentage, and fat mass after the intervention. Lean mass decreased in both groups without between-group differences. The ExPC group maintained significantly lower body fat percentage and fat mass than the Non-ExPC group and showed significantly higher resting oxygen uptake, carbon dioxide production, respiratory exchange ratio, carbohydrate oxidation, and energy expenditure during the intervention. During exercise, the respiratory exchange ratio and carbohydrate oxidation significantly increased in both groups, whereas fat oxidation significantly decreased only in the ExPC group. **Conclusions:** Exercise preconditioning was associated with the maintenance of lower adiposity during short-term ADF combined with concurrent exercise, while differences in substrate utilization were observed under some conditions.

## 1. Introduction

As the global prevalence of obesity continues to rise and the burden of associated metabolic diseases expands, sustainable lifestyle interventions that can effectively manage body weight and metabolic health have become increasingly important [1]. Exercise and dietary interventions are widely used lifestyle strategies for body-weight management and improvement in metabolic health [2,3,4]. In particular, the combination of dietary intervention and exercise is widely used as an effective non-pharmacological approach because it can simultaneously regulate energy intake and increase energy expenditure (EE) [2,5,6,7].

Alternate-day fasting (ADF), which has received increasing attention, is a form of intermittent fasting that induces cyclic changes in energy availability through alternating fasting and feeding days. This dietary strategy may exert favorable effects not only on body composition but also on blood lipids, blood pressure, insulin regulation, and cardiometabolic risk markers [8,9,10]. However, relatively severe and sustained energy restriction may be accompanied by reductions in lean mass [11]. Therefore, combining ADF with exercise is important because this strategy may augment diet-induced reductions in adiposity through increased EE while providing stimuli necessary to preserve muscle function and lean mass. Concurrent resistance and aerobic exercise may provide both mechanical loading of skeletal muscle and oxidative metabolic stimuli, making it an effective strategy for improving body composition and metabolic health [12,13,14].

In a randomized controlled trial involving adults with obesity, participants who combined ADF with aerobic exercise maintained lean mass while achieving greater reductions in body weight and fat mass than those who received either ADF or exercise alone [15]. Similarly, in a randomized controlled trial involving adults with obesity and non-alcoholic fatty liver disease, ADF combined with aerobic exercise reduced body weight, fat mass, and waist circumference; improved intrahepatic fat content and liver enzyme levels; and increased insulin sensitivity without changes in lean mass [16]. In addition, in professional male basketball players undergoing Ramadan intermittent fasting, greater exercise exposure through a higher game frequency was associated with larger reductions in body mass and body fat percentage, suggesting that body-composition responses to fasting may also be influenced by the level of physical activity [17]. Furthermore, a recent systematic review and meta-analysis found that, compared with exercise alone, the combination of intermittent fasting and exercise significantly reduces fat mass and body fat percentage without significant changes in lean mass, suggesting that this strategy may simultaneously promote fat loss and preserve lean mass [18].

However, previous studies combining ADF with exercise have primarily focused on the effects of the intervention itself, whereas the influence of exercise-induced adaptations established before the intervention on subsequent responses remains insufficiently understood. In the present context, “pre-intervention training status” refers to the physiological condition of an animal at the onset of the subsequent intervention, shaped by whether prior regular exercise training has been performed. “Metabolic adaptation” refers to exercise-induced physiological and metabolic alterations, including changes in energy metabolism, substrate utilization, and mitochondrial function [19]. “Exercise preconditioning” refers to the experimentally imposed period of regular exercise performed before the main intervention to establish such adaptations and thereby shape the pre-intervention training status. Thus, these three concepts are related but distinct: exercise preconditioning represents the experimental process, metabolic adaptation represents the physiological changes induced by that process, and pre-intervention training status represents the resulting physiological condition at the onset of the subsequent intervention.

Mechanistically, repeated exercise during the preconditioning period may alter oxidative metabolism and the utilization of carbohydrate and lipid substrates, thereby establishing a metabolic state that differs from that of untrained animals [19,20]. When energy availability subsequently alternates between fasting and feeding, as during ADF, these pre-existing adaptations may influence the relative use of lipid- and carbohydrate-derived fuels [21]. Supporting this concept, prior endurance training has been shown to increase adipose tissue β-adrenergic responsiveness to a subsequent high-fat dietary challenge in mice [22]. Therefore, the metabolic state established before the combined intervention may influence subsequent substrate utilization and energy metabolism responses during repeated fasting–feeding cycles.

Based on this conceptual framework, the present study implemented exercise preconditioning as a distinct phase preceding the subsequent ADF plus concurrent exercise intervention. Previous studies have reported that 4 weeks of regular exercise training can induce physiological and metabolic adaptations, including changes in energy substrate utilization, body composition, and mitochondrial function [20,21]. Based on these findings, a 4-week exercise preconditioning period was adopted, followed by a 7-day intervention combining ADF with concurrent exercise to evaluate early physiological and metabolic responses during the initial fasting–feeding cycles of ADF. Accordingly, this study aimed to evaluate whether exercise preconditioning would modify physiological and metabolic responses to the subsequent combined intervention. We hypothesized that exercise preconditioning would be associated with more favorable changes in body composition, including reduced adiposity and better preservation of lean mass, as well as distinct responses in energy substrate utilization. To test this hypothesis, mice with or without exercise preconditioning were compared with respect to body composition, resting and exercise energy metabolism, muscle function, post-exercise blood biomarkers, and metabolic hormone responses.

## 2. Materials and Methods

### 2.1. Animals and Experimental Design

All experimental procedures were conducted in the animal housing room of the Laboratory Animal Research Facility located in the Heavy Equipment Building at Konkuk University. The study was conducted in accordance with institutional guidelines and was approved by the Institutional Animal Care and Use Committee of Konkuk University (approval no. KU25023).

Sixteen 7-week-old male Institute of Cancer Research (ICR) mice were purchased from Orient Bio Inc. (Seongnam, Republic of Korea) and allowed to acclimatize to the housing environment for 1 week before the experiments. The mice were housed in standard plastic cages containing sterile wood-chip bedding. The animal room was maintained under a 12 h light/dark cycle, with a relative humidity of 50% and a temperature of 23 ± 1 °C. Mice were housed four per cage and received standard laboratory chow and water *ad libitum*.

**Figure 1:**
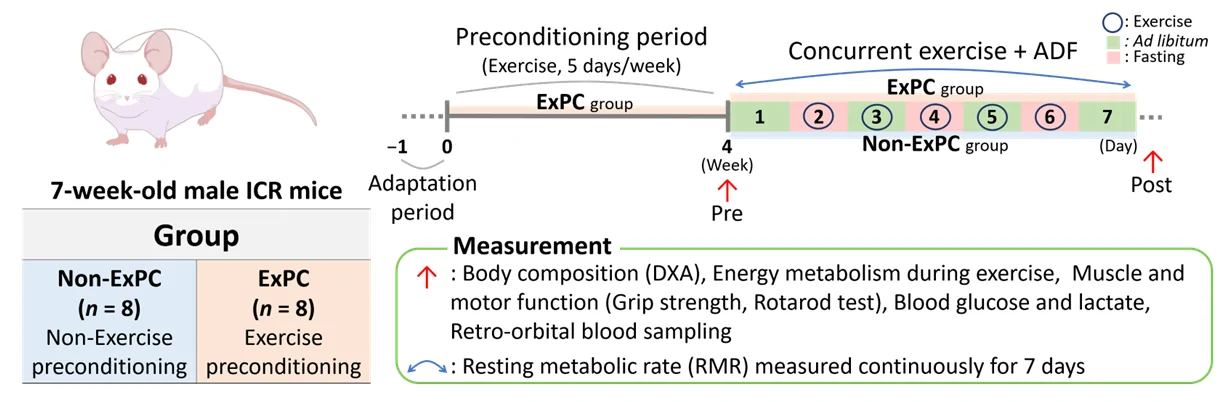
Experimental design. Seven-week-old male ICR mice were acclimatized for 1 week and then randomly assigned to either the Non-ExPC group or the ExPC group. The Non-ExPC group remained sedentary for 4 weeks, whereas the ExPC group underwent 4 weeks of exercise training as an exercise preconditioning phase. Pretests were performed after this 4-week period. Subsequently, both groups received the same 7-day intervention consisting of concurrent exercise and ADF, followed by posttests. RMR was measured daily during the 7-day intervention. ADF, alternate-day fasting; DXA, dual-energy X-ray absorptiometry; ExPC, exercise preconditioning; ICR, Institute of Cancer Research; Non-ExPC, non-exercise preconditioning; RMR, resting metabolic rate.

The sample size (*n* = 8 per group; total *n* = 16) was determined based on sample sizes used in previous rodent studies investigating exercise training, intermittent fasting, and related metabolic outcomes [21,23,24], while also considering the experimental design and the 3Rs principle of minimizing animal use. The four cages were randomly assigned to the two experimental groups, with two cages allocated to the non-exercise preconditioning group (Non-ExPC, *n* = 8) and two cages allocated to the exercise preconditioning group (ExPC, *n* = 8). The 4-week exercise preconditioning program was conducted 5 days per week using a concurrent exercise protocol consisting of resistance exercise followed immediately by endurance exercise. Resistance exercise was performed using a ladder-climbing apparatus for small animals, whereas endurance exercise was performed using a treadmill. A pretest was conducted immediately after completion of the preconditioning phase. Both groups then underwent the same 7-day intervention consisting of concurrent exercise and ADF. During the 7-day intervention, the same 5-day exercise schedule was maintained, with concurrent exercise performed on Days 2–6. A posttest was subsequently performed, and the measured values were compared between the study groups. Figure 1 shows the overall experimental design.

### 2.2. ADF and Exercise Training Protocols

An ADF regimen, consisting of alternating 24 h fasting and 24 h ad libitum feeding periods, was used in this study [23]. During the 7-day intervention period, Days 1, 3, 5, and 7 were feeding days, whereas Days 2, 4, and 6 were fasting days. Mice in each group were individually housed in metabolic chambers throughout the intervention period. On fasting days, food was removed from the chambers, whereas on feeding days, mice were provided with a sufficient amount of standard chow (20 ± 1 g; Charles River Rat and Mouse 18% [Auto], 5L79, LabDiet), providing 3.16 kcal/g of metabolizable energy, with 64.0%, 21.2%, and 14.8% of total calories derived from carbohydrates, protein, and fat, respectively. Water was available ad libitum throughout the experimental period. Food intake was calculated daily from the difference between the amount of chow provided and the amount remaining, and both food intake and body weight were assessed daily at approximately 08:00 throughout the intervention period.

Resistance exercise training was performed using a small-animal ladder apparatus, as previously described, with minor modifications [24,25]. Before the exercise preconditioning period, individual 1RM was assessed using the ladder-climbing apparatus. A plastic tube was secured to the tail using adhesive tape, and the load was adjusted by placing clips and water inside the tube. The initial load was set at 50% of each mouse’s body weight and was progressively increased in increments equivalent to 10% of body weight after each successful climb [26]. The highest load successfully carried to the top of the ladder was defined as the 1RM, consistent with previous ladder-climbing protocols [27]. An attempt was considered unsuccessful if the mouse was unable to continue climbing, fell from the ladder, or failed to reach the top within 1 min. A soft sponge pad was placed beneath the ladder to minimize the risk of injury in case of a fall. When a mouse stopped climbing, the tail was gently stimulated with a sponge to encourage continued climbing. During resistance exercise training, mice performed 10 ladder-climbing repetitions on an 80° inclined ladder. The exercise load was progressively increased from 20% to 50% of the individual 1RM, following the general framework of previously described low-to-moderate-intensity ladder-climbing protocols with minor modifications [24,28]. The procedures for tail loading, injury prevention, and motivational stimulation were identical to those used during the 1RM assessment. After each successful climb, mice were allowed to rest for 2 min in the resting chamber located at the top of the ladder before the next repetition. Immediately after the resistance exercise, endurance exercise was performed using a small-animal treadmill, based on previous studies, with minor modifications [29,30]. Each treadmill session consisted of a 10 min warm-up and 40 min of main exercise, for a total duration of 50 min, at a 15° incline. To provide a low-to-moderate-intensity exercise stimulus throughout the 4-week preconditioning period, the treadmill running speed was progressively increased across the training weeks to 10, 13, 16, and 19 m/min, respectively, based on previous mouse studies using comparable progressive treadmill protocols [29,30]. A mild electrical stimulus (0.4 mA) was applied when necessary to encourage the mice to continue running. The detailed resistance and endurance exercise protocols, including exercise intensity and progression, are presented in Table 1. Training compliance was monitored during each exercise session, and all mice completed the prescribed resistance and endurance exercise protocols.

### 2.3. Body-Composition Analysis

Body composition, including body weight, body fat percentage, fat mass, and lean mass, was assessed using a small-animal dual-energy X-ray absorptiometry (DXA) system (iNSiGHT VET, Osteosys, Seoul, Republic of Korea) [31]. Body composition measurements were performed under standardized conditions, 48 h after the final exercise session and following a 24 h ad libitum feeding period, with a 2 h fasting period before measurement. Water remained available ad libitum during the fasting period.

### 2.4. Assessment of Energy Metabolism at Rest and During Exercise

Energy metabolism at rest was measured during the 7-day combined intervention period using individual metabolic chambers with a volume of 3 L [32]. Each mouse was housed individually in a chamber. Resting energy metabolism measurements were initiated after a 30 min recovery period following exercise to minimize the acute metabolic effects of the preceding exercise bout. VO_2_ and carbon dioxide production (VCO_2_) were continuously monitored, and approximately 22 h of data were included in the daily analysis. The airflow rate in each chamber was maintained at an average of 1 L/min. Respiratory gases were analyzed using a mass spectrometer (ARCO-2000) and a gas-switching system (ARCO-2000-GS-8, both ARCO System, Chiba, Japan). The gas analyzer was calibrated before each measurement according to the manufacturer’s instructions using standard calibration gases. The gas-switching system sequentially sampled each channel for 15 s. During these measurements, eight metabolic chamber channels and two additional air channels were sequentially analyzed, resulting in a sampling interval of 150 s for each chamber. Respiratory exchange ratio (RER), carbohydrate oxidation, fat oxidation, and energy expenditure (EE) were calculated from VO_2_ and VCO_2_ using the accompanying analysis software. RER was calculated as VCO_2_/VO_2_. Carbohydrate oxidation and fat oxidation were calculated assuming a non-protein respiratory quotient using the following equations: carbohydrate oxidation = 4.51 × VCO_2_ − 3.18 × VO_2_ and fat oxidation = 1.67 × (VO_2_ − VCO_2_). EE was calculated using the Lusk equation: EE = 3.816 × VO_2_ + 1.231 × VCO_2_. Nitrogen excretion was not incorporated into the substrate oxidation calculations. VO_2_, VCO_2_, carbohydrate oxidation, fat oxidation, and EE were normalized to body weight for statistical analysis. No specific steady-state criterion was applied. Transient spikes or drops inconsistent with adjacent measurements were considered measurement artifacts and were replaced with the mean of the immediately preceding and subsequent values.

Energy metabolism during exercise was measured in a small-animal exercise chamber using the same mass spectrometry system, based on previous studies, with minor modifications [33,34]. During the exercise metabolism test, mice ran on a treadmill at 16 m/min for 60 min at a 15° incline [33,34]. The entire 60 min exercise period was included in the analysis. The gas-switching system sequentially sampled each channel for 15 s. During exercise measurements, four exercise chamber channels and two additional air channels were sequentially analyzed, resulting in a sampling interval of 90 s for each chamber. The same calculation, normalization, and data-cleaning procedures used for the resting metabolic measurements were applied to the exercise metabolism data.

### 2.5. Grip Strength and Rotarod Tests

Muscle function was assessed using a grip strength meter designed for small animals (JD-A-22, JEUNG DO BIO & PLANT, Seoul, Republic of Korea). For the measurement, each mouse was placed on the metal grid of the device so that all four paws could grasp the grid. The tail was then gently pulled backward at a constant speed until the mouse released its grip. Each mouse was tested three times, and the average value was used as an index of muscle function [35].

Motor function was assessed using a rotarod apparatus designed for small animals (B.S. Technolab, Gyeonggi-do, Republic of Korea), based on previous studies, with minor modifications [36]. After a 1 min acclimation period on the apparatus, the rotation speed was progressively increased by 0.3–0.4 rpm/s. To minimize stress, the test was terminated when the running time reached 120 s, corresponding to 44 rpm. Each mouse was tested three times, and the average running time was used as an index of motor function [37,38].

### 2.6. Blood Glucose and Lactate Measurements

Blood glucose and lactate concentrations were measured from tail vein blood samples collected immediately after the assessment of energy metabolism during exercise. Blood glucose levels were analyzed using a glucose meter (Accu-Chek Performa, Roche Diagnostics, Basel, Switzerland), and blood lactate levels were analyzed using a lactate analyzer (Lactate Pro 2, Arkray, Japan).

### 2.7. Plasma Leptin and Insulin Measurements

Blood samples were collected from the retro-orbital sinus under anesthesia after a 2 h fasting period into heparinized tubes and centrifuged at 2000× *g* for 15 min at 4 °C to obtain plasma. Plasma leptin and insulin concentrations were measured using commercially available enzyme-linked immunosorbent assay (ELISA) kits according to the manufacturer’s instructions. Plasma leptin levels were measured using a Mouse Leptin ELISA Kit (ab100718, Abcam, Cambridge, UK), with plasma samples diluted 10-fold using Assay Diluent A. For insulin analyses, heparinized plasma samples were treated with heparinase prior to ELISA analysis. Plasma insulin levels were measured using a Mouse Insulin ELISA Kit (ab277390, Abcam, Cambridge, UK), with heparinase-treated plasma samples diluted 1:3 using Assay Diluent C. All standards and samples were assayed in duplicate, and absorbance was measured at 450 nm using a microplate reader. Leptin and insulin concentrations were calculated from their respective standard curves after subtraction of the zero-standard optical density and were corrected for the appropriate dilution factors. For plasma leptin and insulin analyses, samples with concentrations outside the quantifiable range of the respective ELISA kits were excluded from the final analysis, resulting in a final sample size of *n* = 6 per group.

### 2.8. Statistical Analysis

Data are presented as the mean ± standard deviation (SD) with individual data points. Statistical analyses were performed using IBM SPSS Statistics version 29 (IBM Corp., Armonk, NY, USA). Normality and homogeneity of variance were assessed using the Shapiro–Wilk test and Levene’s test, respectively. Pre- and posttest data were analyzed using two-way repeated-measures ANOVA, with group as the between-subjects factor and time as the within-subjects repeated-measures factor. Body weight during the 4-week preconditioning period, as well as body weight, food intake, and resting metabolic rate during the 7-day combined intervention period, were analyzed using two-way repeated-measures ANOVA, with group as the between-subjects factor and day as the within-subjects repeated-measures factor. Food intake during the 4-week preconditioning period was measured at the cage level (*n* = 2 cages per group) and was presented descriptively without inferential statistical analysis. For analyses involving more than two repeated measurements, sphericity was assessed using Mauchly’s test, and the Greenhouse–Geisser correction was applied when the assumption of sphericity was violated. When significant main or interaction effects were observed, Bonferroni-adjusted pairwise comparisons were performed. Statistical significance was set at *p* < 0.05. Graphs were generated using GraphPad Prism version 11 (GraphPad Software, Boston, MA, USA).

## 3. Results

### 3.1. Changes in Body Weight and Food Intake During the Preconditioning Period

During the 4-week preconditioning period, body weight significantly increased over time in both groups, with no significant group × time interaction. Post hoc analyses revealed significant increases in body weight in both the Non-ExPC (*p* < 0.001) and ExPC (*p* = 0.001) groups. The ExPC group tended to have lower body weight than the Non-ExPC group (*p* = 0.051). Food intake during the preconditioning period was assessed at the cage level and is presented descriptively in Table 2.

### 3.2. Changes in Body Weight and Food Intake During the Combined Intervention Period

**Figure 2:**
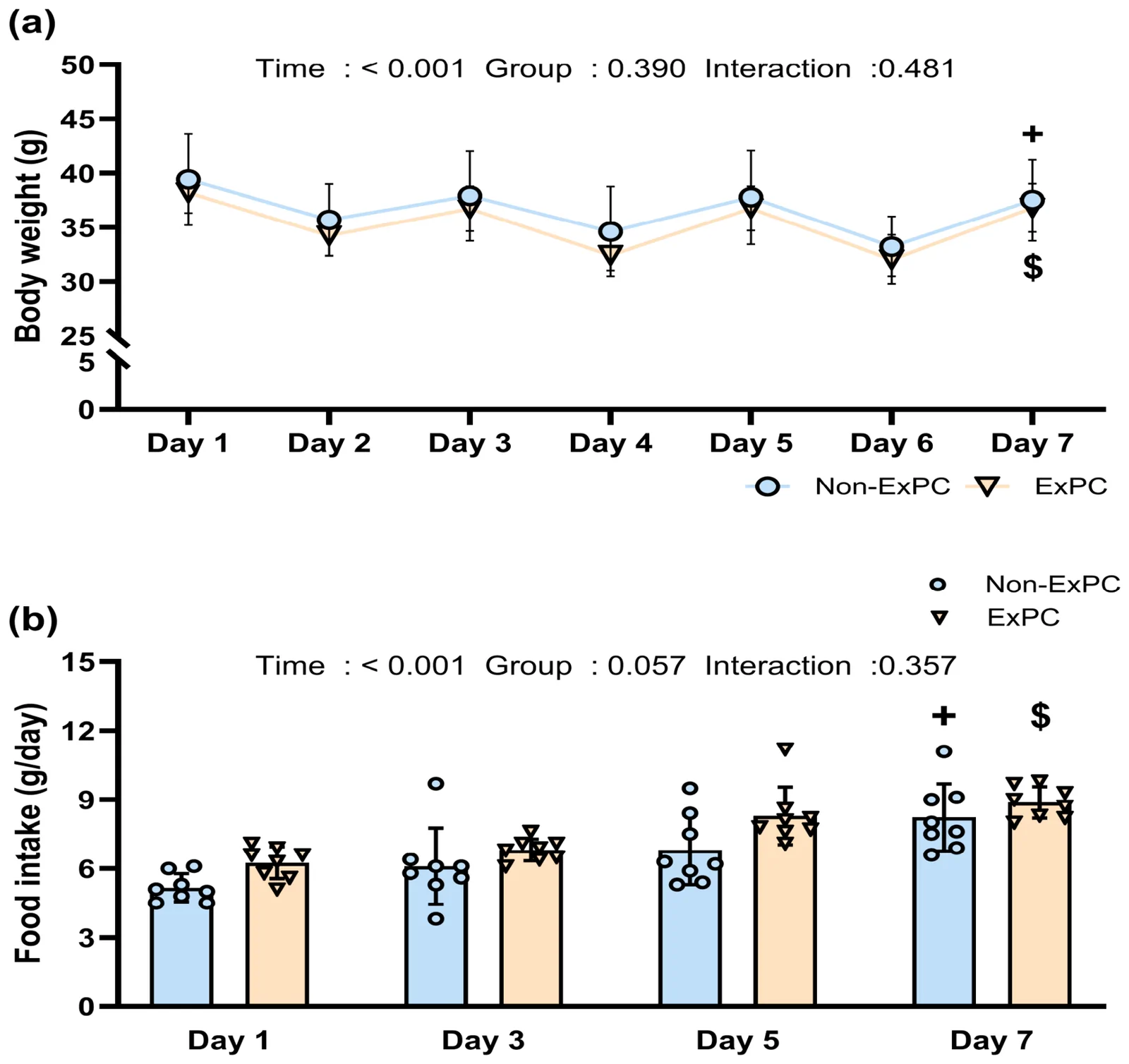
Changes in body weight and food intake during the 7-day intervention. (**a**) Body weight, (**b**) food intake. Values are presented as the mean ± SD (*n* = 8 per group). ^+^ *p* < 0.05 indicates a significant difference within the Non-ExPC group (day 1 vs. day 7). ^$^ *p* < 0.05 indicates a significant difference within the ExPC group (day 1 vs. day 7).

During the 7-day intervention period following the preconditioning period, body weight and food intake were monitored in both groups. For body weight, no significant group × day interaction was observed, whereas a significant main effect of day was detected (*p* < 0.001; Figure 2a). Body weight fluctuated in response to the repeated fasting and feeding cycles during the ADF intervention, and post hoc analyses revealed a significant decrease in body weight from day 1 to day 7 in both groups (*p* < 0.001). No significant between-group difference was observed. Similarly, food intake showed no significant group × day interaction, but a significant main effect of day was observed (*p* < 0.001; Figure 2b). Food intake significantly increased from day 1 to day 7 in both groups (*p* < 0.001), with no significant difference between groups.

### 3.3. Effects of Exercise Preconditioning on Body Composition Following the Intervention Period

**Figure 3:**
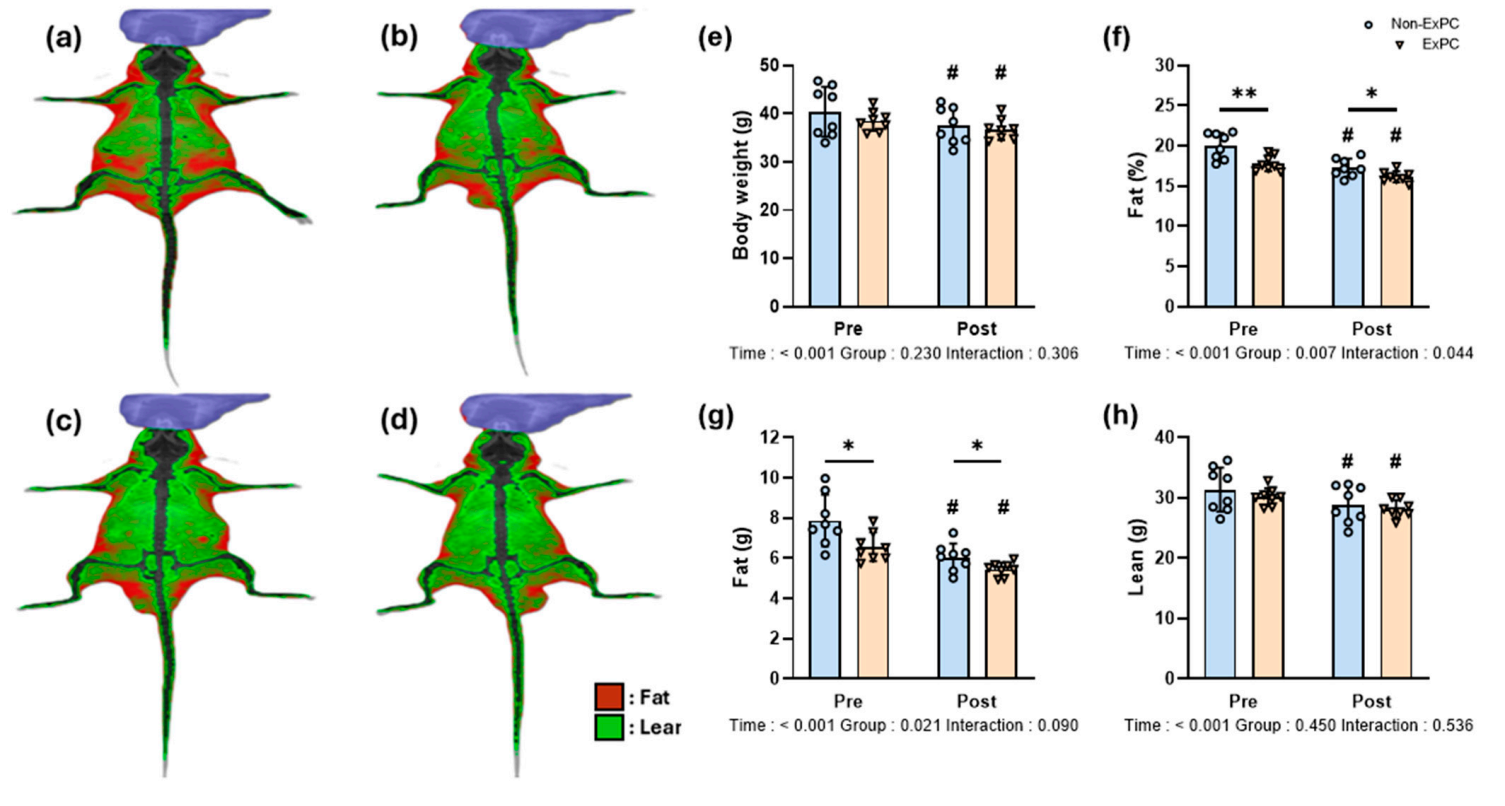
Comparison of pre- and posttest body compositions. Representative DXA images are shown for (**a**) Pre Non-ExPC, (**b**) Post Non-ExPC, (**c**) Pre ExPC, and (**d**) Post ExPC. DXA-derived body-composition data are presented for (**e**) body weight, (**f**) body fat percentage, (**g**) fat mass, and (**h**) lean mass. Data are presented as the mean ± SD (*n* = 8 per group). # *p* < 0.05 indicates a significant within-group difference between pre- and posttest values. * *p* < 0.05 and ** *p* < 0.01 indicate significant differences between groups.

To examine differences in body-composition changes between the Non-ExPC and ExPC groups during the 7-day combined intervention, DXA measurements were performed before (pretest) and after (posttest) the intervention. Representative DXA images are shown in Figure 3a–d, and quantitative body-composition data are presented in Figure 3e–h.

Body weight significantly decreased from pre- to posttest in both groups (Non-ExPC, *p* = 0.001; ExPC, *p* = 0.005), with no significant difference between groups (Figure 3e). Body fat percentage significantly decreased from pre- to posttest in both groups (Non-ExPC, *p* < 0.001; ExPC, *p* = 0.001) and was significantly lower in the ExPC group than in the Non-ExPC group at both pre- and posttest time points (Pre, *p* = 0.007; Post, *p* = 0.028; Figure 3f). Fat mass significantly decreased from pre- to posttest in both groups (Non-ExPC, *p* = 0.001; ExPC, *p* = 0.002) and was significantly lower in the ExPC group than in the Non-ExPC group at both pre- and posttest time points (Pre, *p* = 0.024; Post, *p* = 0.044; Figure 3g). Lean mass significantly decreased from pre- to posttest in both groups (Non-ExPC, *p* = 0.002; ExPC, *p* = 0.012), with no significant difference between groups (Figure 3h).

### 3.4. Resting and Exercise Energy Metabolism According to Exercise Preconditioning

**Figure 4:**
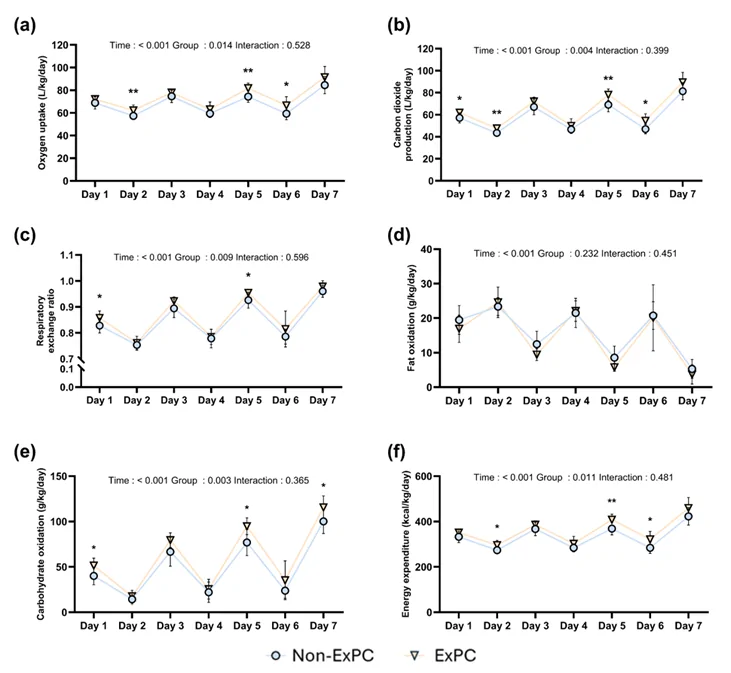
Changes in resting energy metabolism during the 7-day combined intervention. (**a**) VO_2_, (**b**) VCO_2_, (**c**) RER, (**d**) fat oxidation, (**e**) carbohydrate oxidation, and (**f**) EE. Values are presented as the mean ± SD (*n* = 8 per group). * *p* < 0.05 and ** *p* < 0.01 indicate significant differences between groups. EE, energy expenditure; RER, respiratory exchange ratio; VCO_2_, carbon dioxide production; VO_2_, oxygen uptake.

To examine differences in resting energy substrate utilization during the 7-day combined intervention according to exercise preconditioning, daily resting energy metabolism values were analyzed (Figure 4). VO_2_, VCO_2_, fat oxidation, carbohydrate oxidation, and EE are presented as daily cumulative values, whereas RER is presented as daily mean values.

No significant interaction effects were observed for any resting energy metabolism variables during the intervention period (Figure 4). However, significant main effects of time were observed for VO_2_, VCO_2_, RER, fat oxidation, carbohydrate oxidation, and EE (all *p* < 0.001). Significant main effects of group were also observed for VO_2_ (*p* = 0.014), VCO_2_ (*p* = 0.004), RER (*p* = 0.009), carbohydrate oxidation (*p* = 0.003), and EE (*p* = 0.011) but not for fat oxidation.

Post hoc analyses showed that VO_2_ was significantly higher in the ExPC group than in the Non-ExPC group on feeding day 5 and fasting days 2 and 6 (day 5, *p* = 0.009; day 2, *p* = 0.027; day 6, *p* = 0.049; Figure 4a). VCO_2_ was significantly higher in the ExPC group on feeding days 1 and 5 and fasting days 2 and 6 (day 1, *p* = 0.024; day 5, *p* = 0.008; day 2, *p* = 0.005; day 6, *p* = 0.049; Figure 4b). RER was significantly higher in the ExPC group on feeding days 1 and 5 (day 1, *p* = 0.036; day 5, *p* = 0.038; Figure 4c). Carbohydrate oxidation was significantly higher in the ExPC group on feeding days 1, 5, and 7 (day 1, *p* = 0.017; day 5, *p* = 0.011; day 7, *p* = 0.037; Figure 4e). EE was significantly higher in the ExPC group on feeding day 5 and fasting days 2 and 6 (day 5, *p* = 0.009; day 2, *p* = 0.029; day 6, *p* = 0.031; Figure 4f).

**Figure 5:**
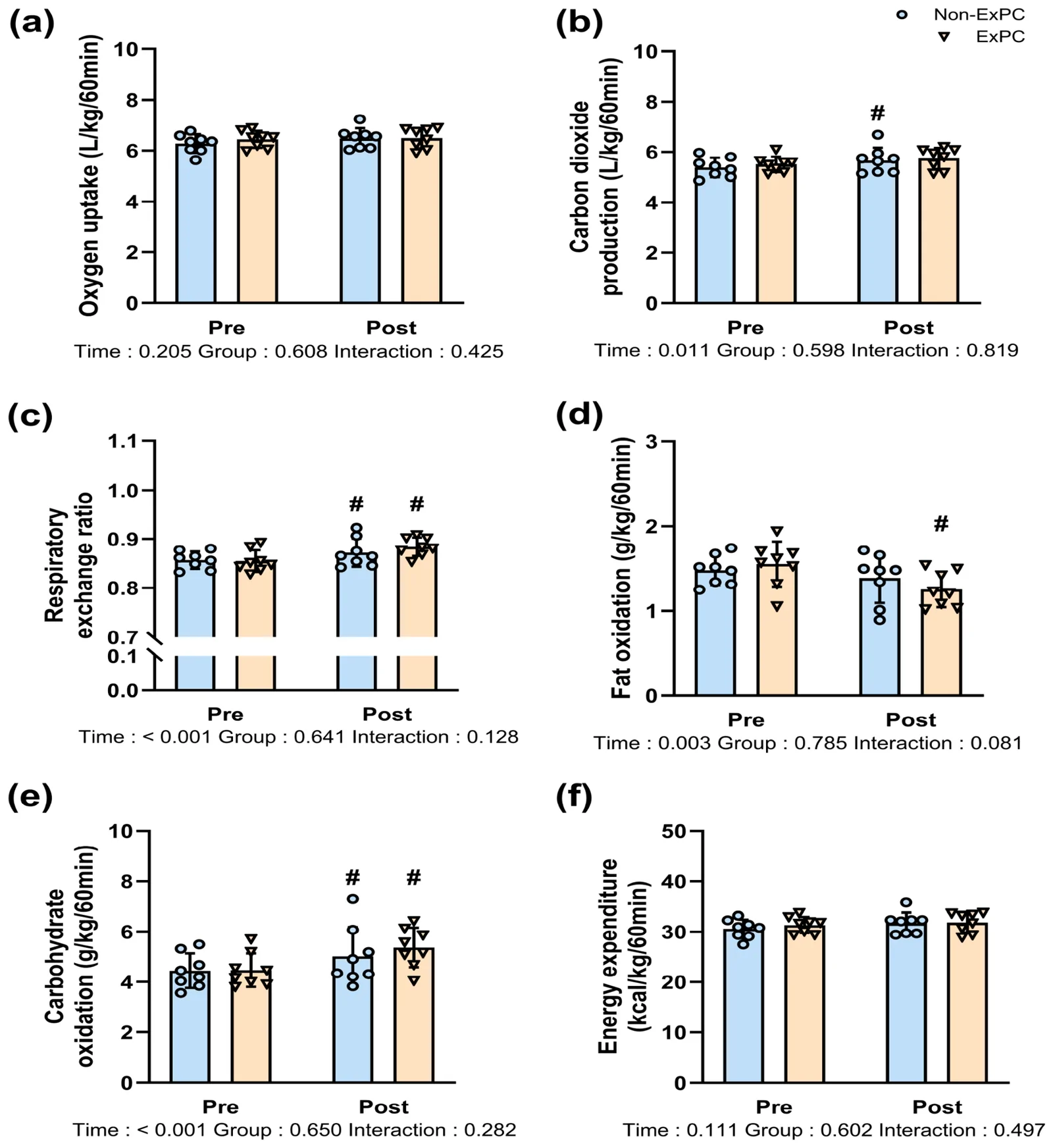
Comparison of pre- and posttest energy metabolism during exercise. (**a**) VO_2_, (**b**) VCO_2_, (**c**) RER, (**d**) fat oxidation, (**e**) carbohydrate oxidation, and (**f**) EE. Values are presented as the mean ± SD (*n* = 8 per group). # *p* < 0.05 indicates a significant within-group difference.

To examine differences in exercise energy substrate utilization before and after the 7-day combined intervention according to exercise preconditioning, energy metabolism during exercise was compared between the pre- and posttest time points (Figure 5).

VO_2_ and EE showed no significant changes from pre- to posttest in either group, and no significant differences were observed between groups (Figure 5a,f). RER and carbohydrate oxidation significantly increased from pre- to posttest in both groups (RER: Non-ExPC, *p* = 0.047; ExPC, *p* = 0.002; carbohydrate oxidation: Non-ExPC, *p* = 0.035; ExPC, *p* = 0.002), with no significant differences between groups (Figure 5c,e). VCO_2_ significantly increased from pre- to posttest only in the Non-ExPC group (*p* = 0.042; Figure 5b), whereas fat oxidation significantly decreased from pre- to posttest only in the ExPC group (*p* = 0.005; Figure 5d). No significant between-group differences were observed for VCO_2_ and fat oxidation.

### 3.5. Muscle and Motor Function with Exercise-Induced Metabolic Responses

**Figure 6:**
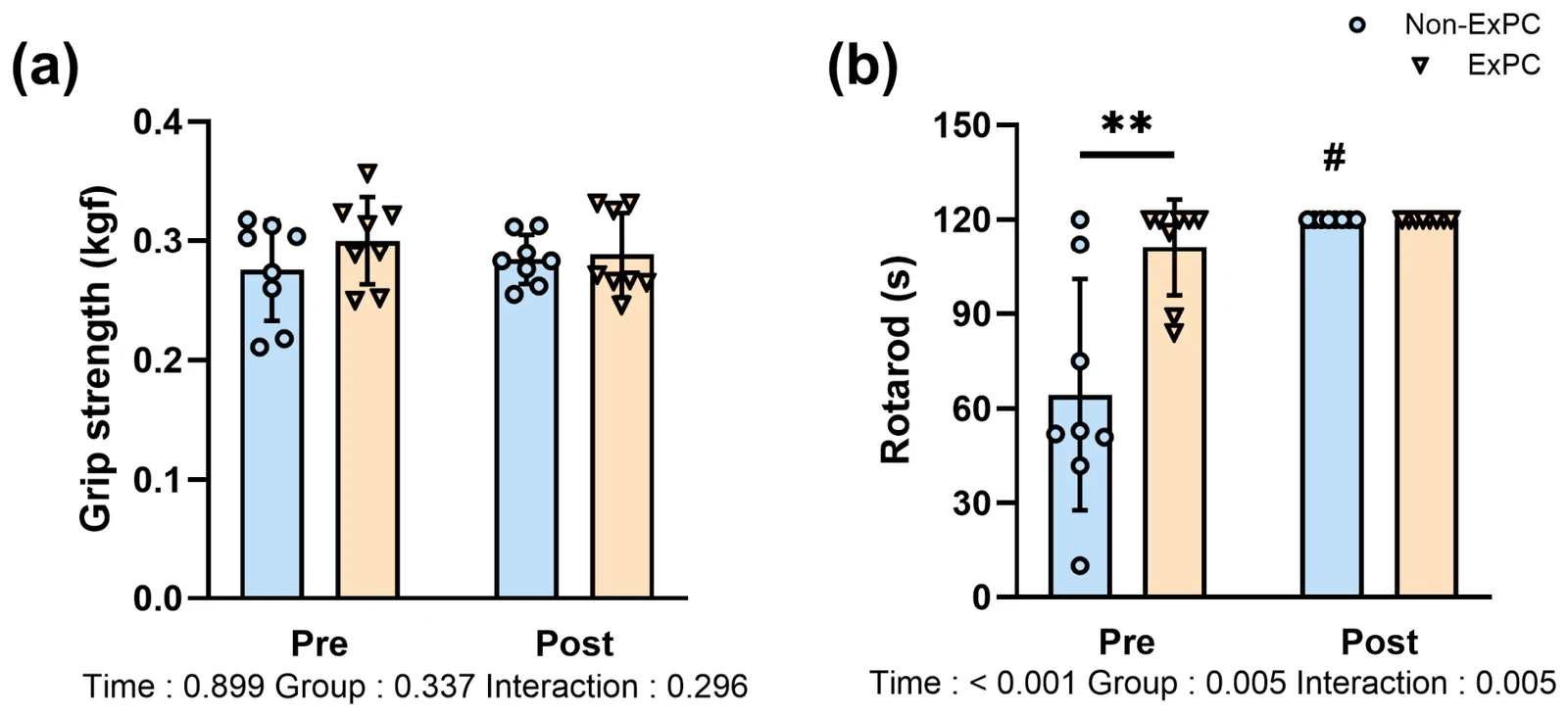
Comparison of pre- and posttest muscle and motor function. (**a**) Grip strength and (**b**) rotarod performance. Values are presented as the mean ± SD (*n* = 8 per group). # *p* < 0.05 indicates a significant within-group difference. ** *p* < 0.01 indicates a significant difference between groups.

To examine changes in muscle and motor function after the 7-day combined intervention according to exercise preconditioning, grip strength and rotarod performance were assessed (Figure 6).

Grip strength showed no significant difference between pre- and posttest values in either group, and no significant difference was observed between groups (Figure 6a). Rotarod performance was significantly higher in the ExPC group than in the Non-ExPC group at pretest (*p* = 0.005). In addition, rotarod performance significantly increased from pre- to posttest in the Non-ExPC group (*p* = 0.004; Figure 6b).

**Figure 7:**
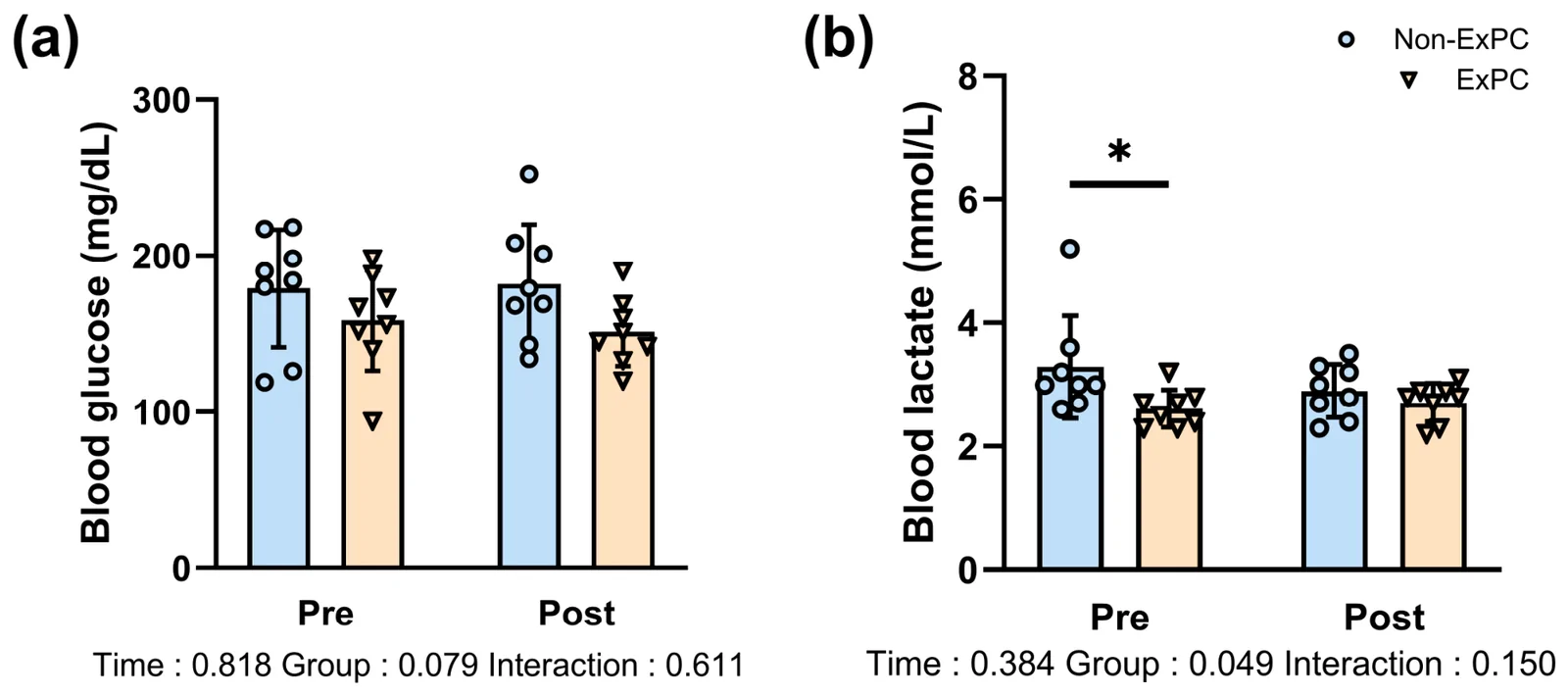
Comparison of pre- and posttest blood glucose and lactate concentrations. (**a**) Blood glucose and (**b**) blood lactate. Values are presented as the mean ± SD (*n* = 8 per group). * *p* < 0.05 indicates a significant difference between groups.

To examine exercise-induced metabolic responses after the 7-day combined intervention according to exercise preconditioning, blood glucose and lactate concentrations were measured from tail blood immediately after the exercise (Figure 7).

Blood glucose concentration showed no significant difference between pre- and posttest values in either group, and no significant difference was observed between groups (Figure 7a). Blood lactate concentration also showed no significant difference between pre- and posttest values in either group; however, it was significantly lower in the ExPC group than in the Non-ExPC group at pretest (*p* = 0.049; Figure 7b).

### 3.6. Circulating Metabolic Hormones

**Figure 8:**
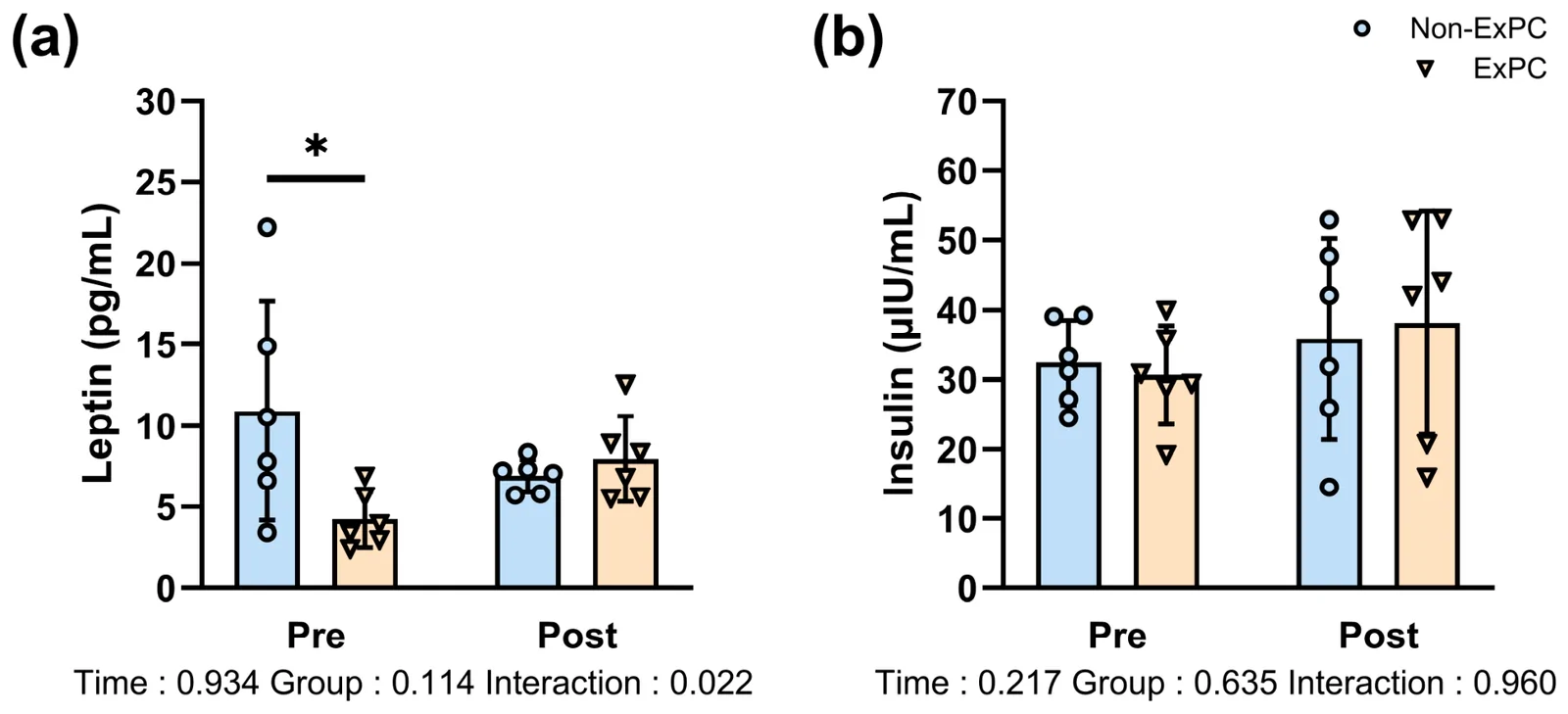
Comparison of pre- and posttest plasma hormone levels. (**a**) Leptin and (**b**) insulin. Values are presented as the mean ± SD (*n* = 6 per group). * *p* < 0.05 indicates a significant difference between groups.

To examine appetite- and metabolism-related hormonal responses during the 7-day combined intervention according to exercise preconditioning, plasma leptin and insulin levels were measured using ELISA kits (Figure 8).

Plasma leptin levels were significantly higher in the Non-ExPC group than in the ExPC group at pretest (*p* = 0.040; Figure 8a). In contrast, plasma insulin levels showed no significant differences between groups or between pre- and posttest values (Figure 8b).

## 4. Discussion

This study investigated the effects of 4 weeks of exercise preconditioning on body composition, energy metabolism, and physiological responses to a subsequent 7-day combined intervention consisting of ADF and concurrent exercise. The ExPC group exhibited lower body fat percentage and fat mass than the Non-ExPC group before the intervention, and these differences were maintained after the combined intervention. In addition, the ExPC group showed generally higher VO_2_, VCO_2_, carbohydrate oxidation, and EE during resting energy metabolism than the Non-ExPC group. In contrast, between-group differences were rarely observed in exercise energy metabolism, muscle function, post-exercise blood biomarker concentrations, and metabolic hormone levels. These findings suggest that exercise preconditioning may contribute to reduced adiposity and a higher level of resting metabolic activity following a short-term combined intervention.

Following the 7-day intervention combining ADF with concurrent exercise of low-to-moderate intensity, both groups exhibited significant reductions in body weight, body fat percentage, and fat mass. These findings are likely attributable to the negative energy balance induced by the combined effects of energy restriction through ADF and increased EE during exercise. They are also consistent with the results of previous studies demonstrating that the combination of ADF and exercise is effective for reducing body fat [39]. However, the ExPC group exhibited lower body fat percentage and fat mass than the Non-ExPC group following the exercise preconditioning period, and these between-group differences were maintained after the subsequent 7-day intervention combining ADF with concurrent exercise. Given that the Non-ExPC group also newly initiated both ADF and concurrent exercise during this period, these findings indicate that the lower adiposity established during the exercise preconditioning period persisted during the early phase of the subsequent combined intervention. Because the group × time interaction was not significant, these findings cannot be interpreted as evidence that exercise preconditioning increased the magnitude of the adiposity response to the subsequent combined intervention. However, the ExPC group entered the combined intervention with relatively lower adiposity, which may have limited the potential for further reductions in body fat [40,41]. Considering this point, the maintenance of lower adiposity together with the additional reductions observed in the ExPC group may suggest that the body-composition advantage established during the preconditioning period persisted into the early phase of the subsequent intervention. In contrast, lean mass decreased significantly in both groups, with no significant between-group differences. ADF, as a relatively intensive dietary intervention, has been reported to induce reductions in skeletal muscle mass alongside decreases in body weight and fat mass, and protein supplementation during fasting periods may not fully attenuate these reductions [11]. In the present study, lean mass measured by DXA also decreased significantly in both groups following the 7-day intervention. However, lean mass assessed by DXA is not equivalent to skeletal muscle mass, and short-term alterations in glycogen and hydration status during ADF may influence DXA-derived lean mass measurements [42,43]. To minimize these potential influences, body composition was assessed under standardized conditions, 48 h after the final exercise session and following a 24 h *ad libitum* feeding period, with a 2 h fasting period before measurement. Although the observed reduction in lean mass cannot be interpreted as direct evidence of skeletal muscle loss, the consistent decrease observed in both groups suggests that lean body composition may change even during a short-term intervention combining ADF with concurrent exercise.

During the 7-day intervention combining ADF with concurrent exercise, analysis of resting energy metabolism revealed that the ExPC group exhibited significantly higher VO_2_, VCO_2_, RER, carbohydrate oxidation, and EE than the Non-ExPC group, whereas no significant between-group difference was observed for fat oxidation. These findings indicate that the ExPC group exhibited generally higher resting energy metabolism and EE during the subsequent alternating fasting–feeding cycles. Notably, on feeding days, the ExPC group exhibited significantly higher carbohydrate oxidation and RER than the Non-ExPC group. Although these differences were not consistently observed on every feeding day, the higher RER and carbohydrate oxidation observed in the ExPC group on some feeding days are consistent with a metabolic pattern characterized by relatively greater carbohydrate utilization after feeding [44]. Substrate oxidation during exercise is influenced by exercise intensity, with changes in the relative contribution of carbohydrate and fat oxidation as exercise demand increases [45]. During exercise, no significant between-group differences in energy metabolism were observed at the pre-intervention assessment. This may have been attributable to the relatively low exercise intensity used during the exercise energy metabolism assessment, which may have been insufficient to fully reveal metabolic differences associated with exercise preconditioning between the groups [46]. After the intervention, both groups exhibited increases in RER and carbohydrate oxidation compared with pre-intervention values, whereas fat oxidation decreased in the ExPC group. Considering that exercise energy metabolism was assessed immediately after the completion of the *ad libitum* feeding period, these changes are consistent with a metabolic state characterized by increased carbohydrate utilization following feeding [47]. However, because postprandial glucose dynamics, tissue glycogen stores, temporal insulin responses, and circulating fatty acid concentrations were not directly assessed, and because the group × time interactions were not significant, these findings do not directly demonstrate enhanced metabolic flexibility or substrate-switching capacity following exercise preconditioning. Collectively, the ExPC group showed relatively greater carbohydrate utilization than the Non-ExPC group under some conditions, which may reflect differences in substrate utilization following exercise preconditioning.

In the assessment of muscle function, grip strength showed no significant changes over time or between groups. These findings suggest that the low-to-moderate intensity of the exercise preconditioning in the present study may not have provided a sufficient stimulus in terms of intensity or duration to induce measurable improvements in maximal strength measures, such as forelimb grip strength [48]. In contrast, rotarod performance, which reflects motor coordination and balance, was significantly higher in the ExPC group than in the Non-ExPC group before the intervention. This finding suggests that the 4-week exercise preconditioning program, consisting of concurrent exercise performed 5 days per week, may have contributed to improved motor function. However, after the intervention, both groups reached the maximum test duration of 120 s, which may have limited the ability of the rotarod test to discriminate between-group differences in performance [49]. Therefore, the absence of between-group differences after the intervention makes it difficult to conclude that the motor-function benefit associated with exercise preconditioning was lost. Exercise-induced changes in circulating biochemical markers can reflect the physiological and metabolic stress associated with exercise, although the specific responses depend on exercise mode, duration, and intensity [50]. Blood glucose concentrations measured immediately after the exercise energy metabolism assessment did not differ significantly between groups at either time point. Given that the exercise test in the present study was performed at low-to-moderate intensity, the absence of between-group differences in blood glucose may reflect the maintenance of glucose homeostasis through a balance between glucose uptake by skeletal muscles and glucose release by the liver during exercise [51]. In contrast, pre-intervention blood lactate concentrations were significantly higher in the Non-ExPC group than in the ExPC group. Although both groups performed the same absolute treadmill exercise protocol, this does not necessarily indicate that the relative exercise intensity was equivalent between groups. Following the 4-week exercise preconditioning period, the ExPC group may have developed greater exercise capacity, such that the same treadmill speed represented a relatively lower workload for these mice. Therefore, the lower blood lactate concentration observed in the ExPC group before the intervention may reflect a difference in the relative metabolic demand imposed by the same absolute workload [52,53]. After the 7-day intervention combining ADF with concurrent exercise, no significant between-group difference in blood lactate concentration was observed, suggesting that the pre-intervention difference in lactate response to the standardized absolute exercise bout was no longer apparent following the common intervention [54].

Plasma hormone analyses were performed using samples collected after a 2 h fast. Leptin concentrations were significantly higher in the Non-ExPC group than in the ExPC group at the pre-intervention time point; however, no significant between-group difference was observed after the intervention. Leptin is primarily secreted by adipocytes, and circulating leptin concentrations are closely associated with fat mass and the energy-storage status of adipose tissue [55]. Therefore, the higher leptin concentrations observed in the Non-ExPC group at the pre-intervention time point may have been associated with the higher body fat percentage and fat mass observed at the same time point. The absence of a significant between-group difference in leptin concentrations after the intervention may reflect an attenuation of the pre-intervention difference in circulating leptin, occurring alongside reductions in adiposity in both groups following the 7-day ADF and concurrent exercise intervention. In contrast, insulin concentrations did not differ significantly by group or time point. Given that insulin was measured at a single time point after a 2 h fast, these findings may indicate similar basal insulin responses between groups under the assessed condition. However, changes in insulin sensitivity or glucose regulation cannot be reliably determined from a single measurement of plasma insulin concentration alone. Therefore, future studies should incorporate additional measures, such as glucose tolerance tests, insulin tolerance tests, and homeostatic model assessment of insulin resistance to more comprehensively evaluate the effects of ADF and exercise preconditioning on insulin sensitivity and glucose homeostasis [56].

This study has several limitations. First, the present study was conducted in young male ICR mice, which limits the direct translational applicability of the findings to humans, particularly individuals with obesity or metabolic disease. In addition, physiological and metabolic responses to ADF and exercise may differ according to species, sex, age, and strain. Therefore, the present findings should be interpreted as preclinical evidence, and further studies in female animals, other strains, older or metabolically impaired models, and ultimately human populations are required to determine their broader clinical relevance. Second, spontaneous physical activity within the metabolic chambers was not directly quantified during the assessment of resting energy metabolism. Therefore, it was not possible to fully distinguish whether the observed between-group differences in VO_2_, VCO_2_, EE, and substrate utilization reflected differences in resting energy metabolism alone or were partly influenced by differences in chamber activity. Future studies should incorporate continuous activity monitoring to more clearly differentiate resting metabolic adaptations from activity-related EE. Third, the molecular mechanisms underlying the observed differences in energy substrate utilization and post-exercise blood lactate responses were not directly assessed. Therefore, it remains unclear whether exercise preconditioning induced molecular adaptations related to mitochondrial oxidative capacity, glucose utilization, lactate transport, or oxidative lactate utilization in skeletal muscle. Future studies should examine mitochondrial function, substrate metabolism-related enzyme activity, and relevant gene and protein expression in skeletal muscle and adipose tissue to elucidate the mechanisms underlying the observed metabolic changes. Fourth, no formal a priori statistical power calculation was performed. Although the sample size was determined based on previous rodent studies with comparable designs while also considering the 3Rs principle, the absence of an a priori power analysis limits certainty regarding whether the study was sufficiently powered to detect all between-group and interaction effects. In addition, although Bonferroni-adjusted pairwise comparisons were applied following significant main or interaction effects, the large number of outcome variables analyzed may still have increased the overall risk of Type I error. Therefore, both non-significant findings, which may reflect insufficient statistical power to detect small-to-moderate effects, and statistically significant findings from individual outcomes should be interpreted cautiously.

## 5. Conclusions

The present findings suggest that the lower adiposity established following exercise preconditioning was maintained during the subsequent short-term intervention combining ADF with concurrent exercise. In addition, differences in substrate utilization were observed between the groups under some feeding and exercise conditions, although these findings do not demonstrate that exercise preconditioning enhanced metabolic flexibility or modified the magnitude of responses to the subsequent intervention. Future studies should conduct longer-term human interventions in adults with obesity or metabolic disease risk, varying the duration of exercise preconditioning, ADF schedules, and exercise intensity to evaluate feasibility, adherence, lean-mass preservation, and metabolic benefits in real-world weight-management settings. Such research may help determine the potential of a phased lifestyle-intervention model integrating exercise preconditioning with ADF and concurrent exercise programs.

## Figures and Tables

**Table 1:** Exercise protocols.

Period	Week	Resistance Exercise		Endurance Exercise	
		(Ladder, 80° Incline)		(Treadmill, 15° Incline)	
4-Week exercise preconditioning period	1	20%	of 1RM	10 m/min	40 min
	2	30%		13 m/min	
	3	40%		16 m/min	
	4	50%		19 m/min	
7-Day combined intervention period	5	50%	of 1RM	19 m/min	40 min

Exercise was performed 5 days per week and consisted of resistance exercise followed immediately by endurance exercise. During weeks 1–4, only the ExPC group performed the exercise protocol as the exercise preconditioning period. During week 5, both groups performed the same concurrent exercise protocol as part of the 7-day combined intervention period. 1RM, one-repetition maximum.

**Table 2:** Changes in body weight and food intake during the preconditioning period.

Variables		Week 1	Week 2	Week 3	Week 4	*p*-Value
Body weight (g)	Non-ExPC	36.3 ± 2.9 ^a^	38.3 ± 3.5 ^b^	39.9 ± 3.9 ^c^	41.2 ± 4.2 ^d^	Time: <0.001 Group: 0.051 Interaction: 0.200
	ExPC	35.2 ± 1.5 ^a^	36.4 ± 1.7 ^b^	37.5 ± 1.5 ^c^	38.7 ± 1.6 ^d^	
Food intake (g/day)	Non-ExPC	5.7 ± 0.4	6.0 ± 0.2	5.3 ± 0.6	4.4 ± 0.6	—
	ExPC	5.2 ± 0.2	5.5 ± 0.6	5.5 ± 0.7	5.3 ± 0.4	

Data are expressed as the mean ± SD. For body weight, *n* = 8 mice per group. For food intake, *n* = 2 cages per group; cage-level food consumption was divided by four to estimate the average intake per mouse. Food-intake data are presented descriptively and were not subjected to inferential statistical analysis. Superscript letters indicate significant differences between time points within the same variable for body weight (*p* < 0.05). SD, standard deviation.

## Data Availability

The data presented in this study are available from the corresponding author upon reasonable request. The data are not publicly available due to institutional data management policies.

## Abbreviations

The following abbreviations are used in this manuscript:

ADF	Alternate-day fasting
DXA	Dual-energy X-ray absorptiometry
EE	Energy expenditure
ELISA	Enzyme-linked immunosorbent assay
ExPC	Exercise preconditioning group
ICR	Institute of Cancer Research
Non-ExPC	Non-exercise preconditioning group
RER	Respiratory exchange ratio
SD	Standard deviation
VCO_2_	Carbon dioxide production
VO_2_	Oxygen uptake
